# Diving into AI? Exploring the Potential for AI to Tackle Complex Water Quality Challenges

**DOI:** 10.1021/acs.est.5c15991

**Published:** 2026-03-28

**Authors:** Edoardo Borgomeo, Luke A. Holmes, Camilla G. Billari, Ioannis Bitsios, Sam Brown, Danielle J. Dickson, Emma Ford, Matt Fry, John Gaffney, Shagun Garg, Marc Girona-Mata, Matteo Giuliani, Nick Hayes, Laura H. Hunt, Andrew Johnson, Milad Latifi, Andrea Marinoni, Harriet G. Orr, Emma Pemberton, Richard Rowan-Robinson, Vidya Samadi, Will Shepherd, Kerry Sims, Simon Spooner, James Tlhomole, Chak-Hau Michael Tso, Tim Williams, Xilin Xia

**Affiliations:** 1 Department of Engineering, 2152 University of Cambridge, Cambridge CB2 1PZ, United Kingdom; 2 41726 Environment Agency, Horizon House, Deanery Road, Bristol BS1 5AH, United Kingdom; 3 Sciens Capital Limited, 34 Bruton Street, London W1J 6QX, United Kingdom; 4 Atmospheric, Oceanic and Planetary Physics and School of Geography and the Environment, 6396 University of Oxford, 9 South Parks Rd, Oxford OX1 3QY, United Kingdom; 5 UK Centre for Ecology & Hydrology, Benson Lane, Crowmarsh Gifford, Wallingford OX10 8BB, United Kingdom; 6 Siemens Digital Industries, Sir William Siemens House, Princess Road, Manchester M20 2UR, United Kingdom; 7 British Antarctic Survey, UK Research and Innovation, Madingley Road, Cambridge CB2 1PZ, United Kingdom; 8 Department of Electronics, Information, and Bioengineering, 18981 Politecnico di Milano, Piazza Leonardo da Vinci, 32, Milano 20133, Italy; CMCC European Institute on Economics and the Environment, Centro Euro-Mediterraneo sui Cambiamenti Climatici, Via Bergognone 34, Milano 20144, Italy; 10 The Rivers Trust, Rain-Charm House, Kyl Cober Parc, Stoke Climsland, Callington PL17 8PH, United Kingdom; 11 WSP, Devonshire Square, London EC2M 4YE, United Kingdom; 12 Department of Computer Science and Technology, 2152 University of Cambridge, 15 JJ Thomson Ave, Cambridge CB2 1TN, United Kingdom; 13 Department of Agricultural Sciences, Clemson University & Artificial Intelligence Research Institute for 30 Science and Engineering, School of Computing, 114625 Clemson University, Clemson, South Carolina 29634-0002, United States; 14 School of Mechanical, Aerospace and Civil Engineering, 7315 University of Sheffield, Mappin Street, Sheffield S10 2TN, United Kingdom; 15 675372 AtkinsRealis, 5 Wellbrook Court, Cambridge CB3 0NA, United Kingdom; 16 UK Centre for Ecology & Hydrology, Library Avenue, Bailrigg, Lancaster LA1 4AP, United Kingdom; 17 295596 Drinking Water Inspectorate, 2 Marsham Street, London SW1P 4DF, United Kingdom; 18 School of Engineering, 67089 University of Birmingham, Vincent Drive, Birmingham B15 2TT, United Kingdom

**Keywords:** AI, water quality, water pollution, drinking-water quality regulation, wastewater infrastructure

## Abstract

Managing risks from water pollution is central to public health, environmental quality, and economic prosperity worldwide. While improvements in water quality have been attained in some parts of the world, much remains to be done to deliver clean rivers, lakes, and seas in line with public interest, changing regulatory landscapes, increasing awareness of risks from pollutants of emerging concern, and climate change. This Perspective explores the potential for artificial intelligence (AI) to help tackle complex water quality challenges. We take a system-oriented approach to define a general pipeline of AI-informed water quality decisions and critically assess the potential of AI to contribute to regulation and decision-making in the context of water quality management. Building on insights obtained from the literature and through a workshop with academics, environmental regulators, industry, and civil society stakeholders in England, we assess the maturity of current AI applications to meet a range of priorities and challenges. While current AI research shows maturity in responding to operational efficiency and modeling and prediction challenges, far less attention has been paid to aligning algorithmic development with user needs and organizational constraints, including the need for trustworthiness and explainability. The full potential of AI to support water quality decisions could be realized through clear institutional processes and accountability frameworks for decision-making. Looking ahead, the development of AI-ready data sets and the availability of clear, open-source examples of AI applications in the water quality domain are potential avenues for supporting wider uptake by regulators and other stakeholders.

## Graphical abstract

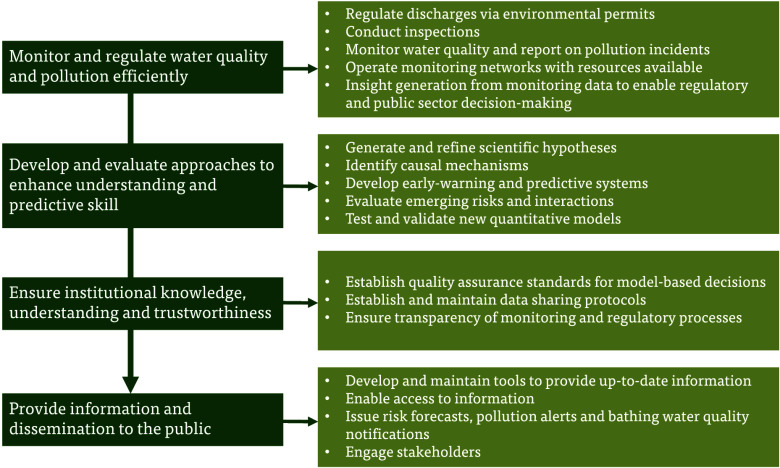

## 1 Introduction

Water quality challenges are numerous and multifaceted, reflecting the broad nature of pollutant sources and pressures globally (Figure 1). Climate change, population growth, aging infrastructure, and complex mixtures of pollutants are all affecting water quality. Human settlements remain a large source of water quality problems. Despite significant expansion of wastewater infrastructure, only 56% of urban wastewater is collected and safely treated globally.^1^ If not well-managed and regulated, then industrial pollutant releases can also be an important source of water quality issues, from contaminants of emerging concern, through to chemicals for which there is established recognition of the risks of exposure, such as per- and polyfluoroalkyl substances (PFAS), and metals. Finally, agriculture has the potential to be a significant source of water pollution, with agricultural runoff, drainage, and livestock farming being responsible for discharge of agrochemicals, organic matter, sediment, and saline drainage into water bodies.^2^ Effective management and regulation are key to limiting the risks of these and other activities.

**Figure 1:**
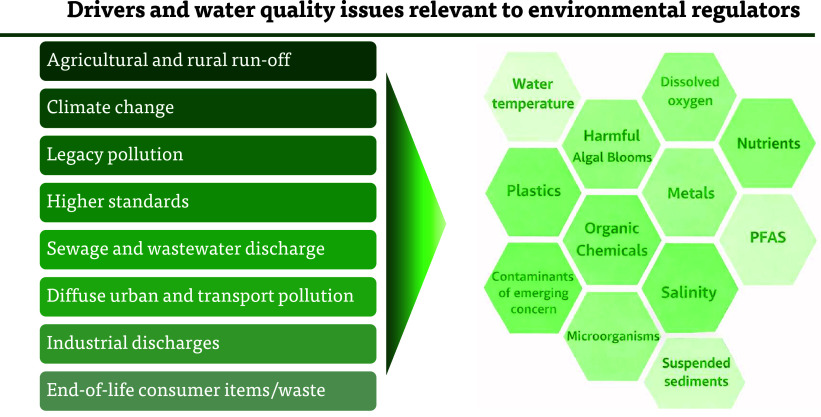
A nonexhaustive list of water quality drivers and variables of relevance to environmental regulators.

Climate change further drives the complexity of water quality challenges. Global climate change is impacting streamflows and the frequency and severity of extreme high and low river flows at continental, national, and regional scales.^3,4^ Water quality challenges may be intensified under extreme high flows due to increased mobilization of sediments and contaminants, or under low flows due to reduced dilution of contaminants and reduced flow velocities.^5^ Climate change also impacts water temperatures, influencing microbial activity and river ecology and related water quality outcomes.^6^

High-income status does not shield countries from water quality challenges.^7^ In the United States, approximately 10% of community water systems experience health-based violations in drinking water quality.^8^ In Europe, while the Water Framework Directive has strengthened water quality monitoring and improved certain parameters, significant challenges persist, for example, increasing awareness of the complexity of chemical pressures on water bodies.^9^

As communities, industry professionals and policymakers become more aware of emerging threats and increasingly demand higher water quality standards, the regulatory landscape becomes more complex. In England, for example, monitoring for Section 82 of the Environment Act 2021 started to come into effect in 2025, requiring “*sewerage undertakers to continuously monitor the quality of the receiving water upstream and downstream of their assets*”, with a minimum of hourly resolution upstream and downstream of storm overflows and wastewater treatment works. To meet this requirement, at least 40,000 new sondes capable of monitoring multiple water quality parameters will be deployed across the country,^10^ making it the world’s densest water quality monitoring network. This very large increase in monitoring capability, albeit for a small number of parameters, paired with the requirement for water companies to make data openly available, may offer significant opportunities to advance modeling and understanding of water quality dynamics.

Scientific advances in the development of large-sample data sets of streamwater chemistry further facilitate uptake of data-driven techniques in water quality.^11,12^ Such shifts provide opportunities and challenges around data integration and interpretation and are one example where recent developments in artificial intelligence (AI) may present a new approach to understanding water quality and improving pollution management.

Recent years have seen rapid advances in the field of AI in hydrology, including flood forecasting,^13^ analysis of urban hydrology signals,^14^ and the creation of large-sample hydrological data sets.^15^ However, most academic research at the intersection of AI and water has focused on algorithm development and testing, with a particular emphasis on water quantity issues—such as flood forecasting—where large data sets are more readily available. To date, AI studies in the field of water quality (i) lack a holistic view that includes the broader institutional and social dynamics that determine water quality decisions and (ii) provide limited examples of AI tools making it through the end of the “pipeline” to operationalization and uptake by decision-makers, from water managers and regulators to citizens.

To address these gaps, this Perspective critically assesses the potential for AI to inform real-world decisions in the heavily regulated context of water quality management. Relative to prior perspectives and reviews on AI-related applications in water quality, we make two contributions. First, we take a broader view of AI, not limiting ourselves to deep learning^16,17^ or machine learning^18,19^ but considering a wider range of AI approaches and related issues, including transparency, ethics, and social impact. Second, we take a system-oriented approach at categorizing potential contributions of AI to water quality, building on insights obtained through a workshop with academics, environmental regulators, industry, and civil society stakeholders in England (see the Supporting Information for more details about the workshop and methodology). The workshop took place in London on April 2, 2025, and was hosted by the University of Cambridge in collaboration with the Environment Agency, an executive nondepartmental public body, sponsored by the UK’s Department for Environment, Food and Rural Affairs. The Environment Agency is responsible for protecting and improving water quality and water resources in England. Although workshop discussions centered on England, the insights and findings presented in this Perspective are relevant to public authorities engaged in water quality regulation, pollution control, and environmental management globally.

## 2 A Framework to Organize AI’s Contributions to Water Quality Management and Regulation

AI holds significant potential to support various stages of water quality management and decision-making process. Given the diversity of actors engaged in decision-making and regulation, this Perspective focuses on the decision and knowledge support needs of environmental regulators, defined as entities tasked with safeguarding water quality (both drinking and environmental) through monitoring, enforcement, permitting, reporting, stakeholder engagement, and guidance, and policy implementation. To structure AI’s potential contributions to decision-making in the context of environmental regulation, we propose a framework that identifies four core regulatory functions and related water quality problems and decisions (Figure 2). Where relevant, the Perspective mentions the potential contributions of AI to decision-making of other actors, such as water utilities, legislators, technology providers, and the general public, among others.

**Figure 2:**
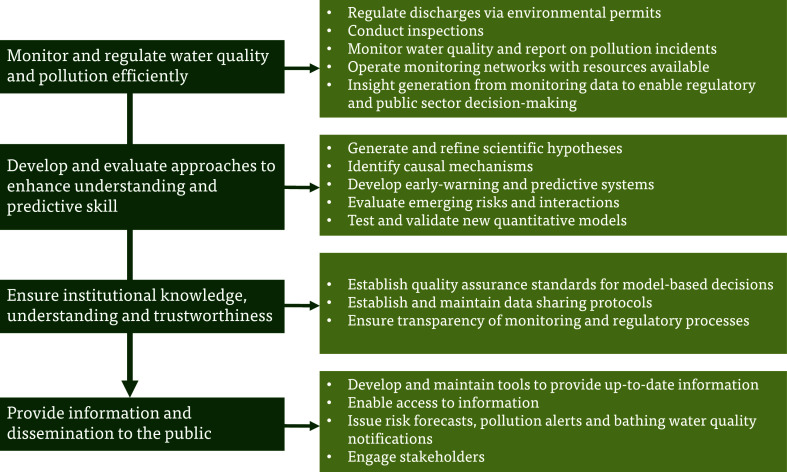
Four key management and regulatory functions (left) and related decision needs (right) define an AI-informed water quality decision-making pipeline.

For each regulatory function (left panel, Figure 2), we (i) identify specific water quality problems and decisions faced by decision-makers (right panel, Figure 2) and (ii) discuss the extent to which AI might be able to assist in addressing them (Table 1). We define AI building upon the taxonomy of AI fields provided in the Artificial Intelligence Playbook for the UK Government^20^ and an exploration of the academic and industry literature. This view of AI encompasses the following fields: neural networks, machine learning, deep learning, speech recognition, computer vision, natural language processing, generative AI, agentic AI, and ethics and societal impact. As shown in the third column of Table 1, some AI fields—particularly machine learning and deep learning—have already been widely deployed to address multiple water quality challenges. In contrast, other fields, such as computer vision, have fewer applications related to water quality.

**Table 1:** Management and Regulatory Functions, Related Water Quality Problems and Decisions, and Opportunities for AI Applications^a^

management and regulatory function	water quality problems and decisions	relevant fields of AI	examples of AI applications or enabling factors
**monitor and regulate water quality and pollution efficiently**	*data harmonization*	machine learning	large-sample, labeled, and meta data-rich AI-ready water data sets^12,15,42^
	*data creation and data filling*	neural networks; machine learning; deep learning	machine learning techniques, including neural networks regression (e.g., decision tree models) to fill incomplete water quality data sets and map contaminants distribution^43^ and leverage other sources of data (e.g., climate forcings) and physical constraints to help fill water quality data gaps;^44^ deep learning to reconstruct river water temperature and dissolved oxygen levels^45^
	*sensor management*	machine learning; deep learning	unsupervised machine learning (e.g., clustering) to optimize sensor placement and activation and to detect sensor faults and drift^46^
	*sampling*	machine learning	machine learning and neural networks to design more efficient sampling routes, timings or locations (e.g., eDNA sampling^47^).
	*anomaly detection*	machine learning	machine learning to detect anomalous data on permit breaches or proximity to permitted values^48^
	*process optimization and control*	deep learning; computer vision	deep learning to develop predictive control policies that optimize energy consumption in treatment processes;^49–51^ computer vision for surveillance and quality control of treatment processes;^52^ deep learning wastewater treatment plant automatic fault detection;^53^ digital twins of wastewater treatment infrastructure^54^
**develop and evaluate approaches to enhance understanding and predictive skill**	*limited insights based on existing data*	neural networks; machine learning	neural networks to predict premature combined sewer overflow (CSO) spills based on CSO chamber depth and other predictors;^55^ machine learning to rank factors responsible for freshwater biodiversity dynamics and stress^56,57^
	*design new water quality treatment and management processes*	machine learning; generative AI	machine learning to design new ways of improving water quality (e.g., new membranes, treatment processes, compounds)^58^
	*predicting data-scarce variables from data-rich surrogates*	machine learning; deep learning	deep learning to predict heavy metal concentrations, antimicrobial resistance burden or emerging contaminant concentrations based on commonly measured parameters and catchment attributes;^17^ LSTM to predict dissolved oxygen from scarce in situ data and large-sample hydrometeorology data;^59^ machine learning for seasonal prediction of algal blooms^60,61^
	*distribution and drivers of water quality levels*	neural network; machine learning	machine learning to predict nitrate and phosphate concentrations and identify key predictors;^62^ spatially weighted neural network regression to integrate remote sensing and in situ data for coastal water quality assessment^63^
	*reliable forecasting of key predictors*	machine learning; deep learning	machine learning based rainfall nowcasting to improve bathing water quality forecasts;^64–66^ class-imbalance learning of bathing water quality^67^
	*scenario exploration*	machine learning; generative AI	digital twins of rivers and aquifers to explore impact of land-use and climate scenarios and help plan strategic interventions to protect the environment^68,69^
	*prediction at unmonitored locations*	deep learning	deep learning applied to large-sample data sets to predict water quality at unmonitored locations^59^
	*incorporation of complex spatial data sets into predictive models*	neural network; machine learning	convolutional neural networks or graph neural networks to predict surface and groundwater quality^17^
	*prediction of antimicrobial resistance in water bodies*	machine learning; deep learning	machine learning to predict antibiotic-resistant bacteria (ARB) and antibiotic-resistance genes (ARGs) and support interpretation of genomic data^70^
**ensure institutional knowledge, understanding and trustworthiness**	*data sharing protocols*	machine learning; natural language processing	AI-enabled data rescue and standardization^71^
	*explainability*	deep learning; machine learning;	data points in larger data sets are required to remain confidential (e.g., monitoring data on water quality for private drinking water supply). These data cannot be made findable, accessible, interoperable and reusable (FAIR) because of potential confidentiality and security implications, thus there is a need to find alternative data sharing protocols.
	*uncertainty analysis*	machine learning	process guided deep learning to improve interpretability of AI-based water quality predictions^72^ and tools in explainable AI (e.g., feature attribution techniques)^73^
	*legal and regulatory procedures*	ethics and societal impact	probabilistic machine learning (e.g., Gaussian process regression) to quantify uncertainty in water quality predictions^73^
	*coupled human and AI decisions*	generative AI, ethics, and societal impact	frameworks to understand accountability and regulatory compliance of AI^34^
	*concept explanation*	generative AI	explore ethical and societal considerations, including (i) use of LLMs as autonomous agents for real-time water quality decision-making and/or as part of multiagent systems coupled of AI with existing mechanistic models and human knowledge (heuristics, expert judgment)^35^ and (ii) potential for human in the loop frameworks for water quality decisions, building upon similar debates in environmental and climate sciences^74^
**provide information and dissemination to the public**	*working with citizen scientists*	natural language processing	sequential information retrieval and concept explanation to facilitate learning of new or emerging themes and research methods^75^
	*establishing two-way communication channels*	natural language processing	explore AI’s potential (e.g., natural language processing, neural networks and deep learning) to pool, store, interpret observations, data, and other inputs from citizens^76^
	*uncertainty communication and public trust in AI*	ethics and societal impact	establish AI-based digital assistants to increase engagement and communication with citizens (e.g., England’s Environment Agency use of Hello Lamp Post for Water Watch), which allow citizens to access readily available information on Bathing Waters and provide citizen science observations and feedback^41^
	*bridging the digital divide and ensuring accessibility*	generative AI	conveying uncertain information is challenging, and there is no research, minimum threshold error, or agreed standard on conveying uncertainty in AI-generated water quality information. Any false alarm is likely to further undermine public trust in AI and risks damaging credibility of water quality science
	*ethics and governance in AI-citizen Interaction*	ethics and societal impact	develop AI for low-bandwidth settings (e.g., SMS alerts, voice assistants in local languages), and visual, audio, or offline interfaces to include digitally underserved communities

a For a definition of the relevant fields of AI identified in this table, see ref 20.

Table 1 does not provide a comprehensive comparison of AI techniques vis-à-vis conventional methods; rather, it shows the potential of AI to respond to some specific decision and knowledge needs. Comparisons with conventional approaches are available in existing review articles, including. refs 17,21,22 . These reviews describe some important advantages of AI approaches over conventional statistical or process-based models for water quality problems, particularly in their ability to learn complex, nonlinear relationships from heterogeneous and high-dimensional data, fill temporal and spatial gaps, and scale predictions across broad spatiotemporal extents. These reviews also highlight challenges related to AI, including overfitting without sufficient data, sensitivity to heterogeneous monitoring coverage, and lack of interpretability and generalizability.

### Monitor and Regulate Water Quality and Pollution Efficiently

Monitoring water quality and regulating pollution efficiently are core functions of environmental regulators. This involves operating and enhancing water quality monitoring and modeling systems under resources available, targeting and conducting inspections based on priority and risk, and controlling discharges through environmental permits while overseeing and enforcing compliance. Resource constraints—economic, human, and time-related—combined with data and model uncertainty and the expanding scope of regulatory requirements (e.g., monitoring more parameters at higher temporal and spatial resolution) heighten the value regulators place on operational efficiency. In this context, efficiency means delivering high-quality monitoring and regulatory oversight while ensuring value for money.

Several existing AI applications respond to decision needs related to monitoring and regulating water quality and pollution efficiently. For example, confronted with systemic data gaps, regulators could leverage recent experiences aimed at the harmonization of hydrologic and climate data,^15,23^ complemented by the use of generative models for data generation.^24,25^ Similarly, AI methods such as computer vision can provide a more cost-effective approach to conduct real-time anomaly detection (e.g., wastewater treatment performance^26^).

### Develop and Evaluate Approaches to Enhance Understanding and Predictive Skill

To inform their decisions, regulators need an understanding of how water quality evolves under the combined influence of global change, local drivers, and human activities. Achieving this understanding often requires formulating new scientific hypotheses to explain observed trends in the data and identifying the causal mechanisms behind these trends. For example, to enable adaptive regulation and effective management actions, key priorities for regulators include (i) projecting water quality trends under alternative climate change and land-use or management scenarios; (ii) modeling which factors are more significant in influencing storm overflows and contaminant dilution at different locations; (iii) predicting the occurrence and modeling the impact of contaminants of emerging concern; (iv) quantifying the role of water quality on ecosystem health.

Insights from these efforts underpin the development and refinement of predictive systems capable of modeling water quality dynamics, producing quantitative estimates of future water quality conditions across space and time, and discerning the impact of multiple water quality stressors in ecology. AI can be deployed to support all of these tasks, as demonstrated by recent applications in water quality and similar fields. For example, machine learning has been deployed to predict the occurrence of PFAS in groundwater across the United States^27^ or understand the processes governing PFAS removal in membrane-based treatment systems.^28^ In other fields, generative AI was used to generate scientific hypothesis,^29^ capture complex and nonlinear dependencies among key drivers of heatwaves,^30^ and provide timely, spatially detailed forecasts of extreme floods in ungauged basins.^13^ These advances—integrated with the expertise and knowledge of domain experts—can potentially transform water quality modeling and forecasting and its contribution to regulatory design, monitoring, and implementation. These advances can also underpin new risk assessment frameworks for wastewater and water technologies (e.g., industrial water risk assessment to identify pollution sources and mitigation strategies to comply with regulations,^31^).

### Ensure Institutional Knowledge, Understanding, and Trustworthiness

All public decisions made with respect to water should be transparent, accountable, and easily interpretable.^32^ In the field of water quality, environmental regulators and decision-makers are therefore expected to ensure institutional knowledge, understanding, and trustworthiness of the tools used to support decisions. Fulfilling this function is particularly challenging when deploying emerging technologies such as AI for which knowledge, understanding, and trustworthiness may still be limited.

A trustworthy AI system can help mitigate social and political risks—such as those stemming from real or perceived bias (e.g., disproportionately targeting inspections in low-income areas due to sparse data coverage), inaccurate forecasts or excessive false alarms, and vulnerability to manipulation (e.g., utilities altering reported data to influence model outputs).^33^

In the context of AI, the need for transparency and trustworthiness highlights two interrelated questions of relevance for regulators. First, can AI help to improve the transparency and trustworthiness of regulatory decisions regarding water quality? Little research has explored the extent to which AI can improve trust in regulatory decision-making.^34^ This is despite the growing evidence suggesting that large-language models (a form of generative AI) can be used as agents to conduct several tasks autonomously, such as building models of environmental systems or simulating the behavioral responses of alternative stakeholder groups to environmental regulations.^35^

Second, what are the opportunities to enhance the trustworthiness and transparency of AI models used for water quality monitoring and management? The black box nature of many AI models is a significant bottleneck to their widespread application in regulated industries, where clear procedures to determine accountability for decisions are paramount. However, recent advances in explainable AI might provide a way forward for gradually improving trust in AI and formal institutional uptake.^36^ Explainable AI consists of a set of techniques and workflows to interpret the output of AI models such as machine or deep learning models.^37^ These approaches have already demonstrated potential in interpreting results from data-driven hydrological models.^38^ Application of these approaches is considered paramount to support model transfer and uptake for environmental management and is a key step for best practice approaches in the field.^21^

### Provide Information and Dissemination to the Public

Citizens and the natural environment are the ultimate beneficiaries of any regulatory decision made with regard to water quality. AI has clear potential to provide tailored and more timely information and insights to the public (e.g., potential economic benefits for water users under hydroclimatic forecasts^39^) and improved citizen engagement by facilitating citizen science (e.g., estimating snow-covered area using crowdsourced images,^40^). For example, in England, the Environment Agency is piloting AI-based chatbots by deploying the Hello Lamp Post AI platform to provide additional means to engage and communicate across several designated swimming sites in England (Bathing Waters). Through QR signage, citizens can access real-time information on water quality including microbiological insights relevant to swimmers’ health, while also answering a series of questions to support data collection and monitoring activities.^41^ AI tools have the potential to support broader efforts aimed at engaging with individuals and communities, such as farmers, whose actions can have a direct impact on water quality. AI tools would be a component of the overall systems required to engage with these stakeholders and bring about transitions in the built environment and land management required to enhance water quality.

## 3 System-Level Assessment of Enablers and Constraints

The previous section identified four key management and regulatory functions and related decision and knowledge needs in the field of water quality. The previous section also presented select examples of the contributions and potential benefits that AI might provide. Potential benefits include processing large amounts of different data sets from different sources quickly, detecting anomalies, and also supporting a range of workplace procedures. However, despite the promising developments identified above and the potential benefits of AI often mentioned by researchers, consultants, and the media, effective and transparent adoption of AI technologies within the water sector still faces various constraints. These constraints originate from the obvious notion that regulatory agencies involved in water quality management need to first and foremost concentrate on fulfilling statutory requirements such as ensuring public health and environmental protection^77^ while developing, testing, and integrating new approaches.

Alongside these constraints, which may influence institutions’ ability to achieve value with AI, a range of other—and arguably more important—issues need to be considered. Contextual factors, water system characteristics, organizational incentives, and specific actors or entities within these institutions collectively interact to influence the uptake of AI, the speed and transparency of adoption and utilization, and the realization of AI-related benefits. Additionally, the perception of the technology itself—including perceived benefits, trust, and familiarity—also affects the adoption and diffusion of AI. Collectively, these factors define a system that influences the adoption of AI in water quality (Figure 3). The system view in Figure 3 builds upon the systems thinking literature^78^ and research on technology adoption.^79^ In systems thinking, an organization and its external context are understood as a complex, interconnected whole, composed of interdependent elements rather than isolated components.^80^ The interaction between the external and organizational context with the technology context influences uptake and also the ability of organizations to realize the benefits and public value of a given technology. Here, we highlight 10 core enabling factors that emerged more prominently during the workshop.

**Figure 3:**
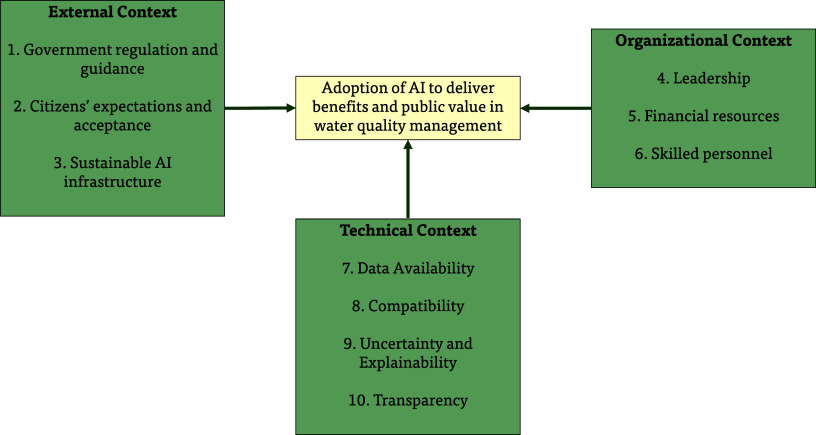
Framework for analyzing uptake and diffusion of AI technologies in water quality, with main enabling factors under each context aspect.

Government-wide regulation and guidance are central to the external context and will likely be the most significant drivers of AI adoption in water management (factor #1). Their influence stems from the public sector nature of most water-related decisions and the obligation of regulatory agencies to comply with directives, legislation, and policy frameworks. Government guidance may include, for example, instructions on how to integrate AI into organizational processes along with clear procedures to ensure transparency, accountability, and ethical oversight. This is particularly relevant in cases where decisions—such as issuing a swimmer safety alert—are made entirely by AI systems or are partially informed by AI agents. In Australia, for example, the Policy for the Responsible Use of AI in Government sets mandatory expectations on the use of AI by public sector agencies, including those mandated to regulate water quality.^81^

Citizens’ expectations and acceptance of AI-informed decisions on water quality are key components of the external context (factor #2). Social and political issues, including democratic legitimacy, overall trust in institutions, and politicization of environmental evidence are other important aspects that may influence citizens’ expectations and acceptance. Research on human-centric approaches to designing and implementing AI solutions will be crucial to understanding perceptions among citizens regarding acceptability.^74,82,83^ In the context of air quality, human-centric and personalized approaches show potential to inform personal health-related decisions.^84^ Finally, the external context also includes sustainable AI infrastructure (factor #3). Data centers have energy, infrastructure, and water requirements and may have significant undesirable localized impacts on water resources, and on key water quality parameters such as water temperature. Regulators will be expected to identify any potential negative impact arising from their use of data centers, including reducing water and carbon footprints as part of wider sustainability measures and related mitigation measures.

The organizational context influences the pace of innovation in the water sector,^77^ and this is also true for AI. Reputational management and low confidence in new approaches will likely initially limit the use of AI to carefully selected lower risk tasks, such as supporting either the preparation of water quality reports following an extreme event or organizational performance reviews. To shift application to broader uptake, we identify three key enabling factors: leadership (factor #4), financial resources (factor #5), and skilled personnel (factor #6). Establishing a culture of innovation with appropriate safeguards and guidelines can support AI adoption. Guidance for government departments and public sector organizations provides a common foundation upon which to build this culture. For example, the UK Government’s AI Playbook^20^ defines the principles of safe, responsible, and effective use of AI, while within organizations, the uptake of AI is beginning to feature explicitly in ambition statements on innovation (e.g., refs 85,86 ).

The technical context is also important in determining the uptake. Data remain one of the largest bottlenecks to broader AI adoption in water quality (factor #7). Globally, data issues include uneven distribution of data across space, with 71% of water quality data globally from monitoring stations located either in North America or Europe, absence of long-term data sets required to analyze trends, low to no data points on emerging contaminants, and strong bias toward surface water data.^87^ While integration of water quality data in large-sample hydrology data sets^88^ and innovations in data collection can help bridge the data gap, sustained investments in in situ monitoring will be required to advance AI uptake, particularly across Africa, Latin America, and parts of Asia and Oceania where there are currently no or very limited observational records.

Compatibility, transparency, and explainability are additional relevant factors in terms of AI technology uptake. Regarding compatibility (factor #8), security is an issue of high importance. For example, AI models developed by third-party technology providers will need extensive vetting before implementation and potential inclusion in regulatory decision-making. Issues concerning uncertainty and explainability in AI tools also emerge as important aspects in influencing uptake (factor #9). Uncertainty around how an AI model has been trained, challenges around recording and interpreting any AI-informed decisions, and inherent bias in training sets need to be fully communicated and described for AI tools to contribute to regulatory decisions. Finally, in terms of transparency, ethical and individual rights issues emerge as important factors in influencing uptake (factor #10). Ethical issues include risks related to AI introducing bias or inequality in water quality decisions and outcomes, while individual rights issues encompass processing of any personal data in an AI system. Algorithm registers are one approach to systematically documenting how AI tools are used by public authorities to enhance transparency. In The Netherlands, public bodies can publish information about algorithms and AI tools they use to fulfill their mandates in the Algorithm Register of the Dutch Government.^89^ This approach is intended to increase transparency and trust in government decisions informed by AI, including water-related decisions, such as water body monitoring. Research on compatibility, transparency, and explainability is nascent, and this severely impacts operationalization within a heavily regulated policy area such as water quality. Research on the topic of transparency in the use of AI is particularly scarce, and this is a significant gap to overcome to enable further engagement with and future uptake of AI.

## 4 Outlook

The potential for AI to drive innovation in water quality regulatory and management decisions is clear, and several AI-based approaches already demonstrate the potential to support water quality decision-making (Table 1). Despite this potential, in Section 3, we identified several factors that need to be considered and addressed in research and practice alike to further promote and extend the uptake of AI in this field. As research expands new frontiers in the use of AI for water quality management, it is useful to look beyond the core focus on algorithm testing and development. This Outlook Section identifies two considerations that may support more effective and transparent integration into regulatory decision-making. While these considerations are most directly relevant in contexts with relatively mature monitoring systems and regulatory frameworks, they may also be informative for countries with emerging or data-limited water quality regimes, where they could contribute to a phased strengthening of public sector capacity and governance related to AI-enabled water quality management.

### Consideration #1: Water Quality Infrastructure at Regional, National, and Transnational Scales

Standardized data infrastructure helps to streamline data ingestion and analysis within and across research groups and agencies. In the case of deep learning applications, data availability and quality are regarded as the main bottleneck to further advances and discoveries.^17^ In turn, limited data uptake is a major bottleneck to enhancing the performance and trustworthiness of AI for water quality decisions.

Increased coordination among actors involved in data generation (e.g., water utilities, civil society organizations, environmental regulators, and researchers) contributes to the advancement of water quality data infrastructure. In this context, two actions have been identified in the literature as influential. First, development of consistent data standards, formats, and protocols for FAIR (Findable, Accessible, Interoperable, and Reusable) data^90^—while still safeguarding sensitive information. This includes developing data models that clearly define how water quality data and related models are organized, validated, and exchanged, so as to facilitate its use by AI systems. Second, development of protocols and data pipelines to access and utilize third-party data, including from citizen scientists. Developments in large-sample data sets for water quality^11,12^ could be further pursued to enhance compatibility and standardization.

AI can be used to speed up the production of large-sample data sets, while good-quality, AI-ready data sets are key to advances in AI applications. For example, machine learning-ready data formats that also capture metadata make them easier to use for AI/ML pipelines,^91^ such as RO-Crate^92^ and Croissant-ML.^93^ Early steps toward a common data infrastructure include the UK’s Catchment Systems Thinking Cooperative (CaSTCo) efforts to develop a national framework that supports decision-makers, scientists, and communities in using citizen science data.

### Consideration #2: Shared Understanding between Researchers and Practitioners on Standards for Model-Based Decision-Making and Forecasting

The extent of shared understanding between researchers and practitioners regarding relevant standards and practices has been linked to the effectiveness of model-based decision-making and forecasting in environmental management.^94^ Issues commonly discussed include the selection of metrics, model performance, thresholds, treatment and communication of uncertainty, procedures, and accountability. Traditional water quality metrics could be combined with more innovative metrics, such as financial risks or economic values of fines,^95^ to predict more decision-relevant variables for certain stakeholder groups, such as water utilities.

Collaboration between AI and model developers, regulators, and other decision-makers contributes to establishing a consensus on what is achievable and appropriate in this space. Improved clarity on these issues will (i) inform benchmarking exercises; (ii) guide the identification of AI tools that are fit-for-purpose in regulatory decision-making; and (iii) support the development of appropriate ethics and risk management procedures. The latter has been highlighted as particularly relevant for enabling the responsible use of AI-based approaches in water-related applications.^68^ Enabling knowledge transfer between disciplines in the water quality and AI fields would ensure future frameworks and standards used for decision-making take into account recent innovations. It would shed light on areas for which AI might not be required or fit-for-purpose, because of costs, the need for ethical judgments, or transparency and high levels of trust. This suggestion to review frameworks and standards echoes calls for improved approaches to benchmarking flood hydrology models to better guide their uptake in practice.^94^

Work in this area requires research by social scientists as much as AI engineers and computer scientists. AI engineers and computer scientists can provide quantification of the reliability and limits of AI tools in addressing decision-making needs. Social scientists can help regulators understand their attitudes toward AI-based outputs, related uncertainties, and the role of risk preferences in influencing AI uptake in decision-making. Social scientists can also help understand public attitudes to AI-based outputs and help develop participatory modeling pipelines that do not just enhance public understanding but also actively inform decision support systems.^96^

To conclude, the application of AI in water quality remains at a relatively early stage. This Perspective identified a wide range of potential applications spanning data generation, operational decisions, and public engagement. To capture this potential, it is important for researchers to complement their traditional focus on technology testing and development with a system-level view that considers how external and organizational factors influence technology adoption. Interdisciplinary collaboration and clear and open-source examples of AI applications in the water quality domain will be critical to ensuring technical innovation and trust.

## Supplementary Material



## Notes

Details about the workshop and methods used to develop this perspective article (PDF)
