# Supplementary material for: Diving into AI? Exploring the Potential for AI to Tackle Complex Water Quality Challenges
Source: Environ Sci Technol. 2026 Mar 28;60(14):10405–16. doi: 10.1021/acs.est.5c15991 (PMC13085810; doi:10.1021/acs.est.5c15991)
Supplement: Supplementary file 1 [file es5c15991_si_001.pdf]

Supporting information

Diving into AI? Exploring the potential for AI to tackle complex water quality challenges

Edoardo Borgomeo; Luke A. Holmes; Camilla G. Billari; Ioannis Bitsios; Sam Brown; Danielle J. Dickson; Emma Ford; Matt Fry; John Gaffney; Shagun Garg; Marc Girona-Mata; Matteo Giuliani; Nick Hayes; Laura H. Hunt; Andrew Johnson; Milad Latifi; Andrea Marinoni; Harriet G. Orr; Emma Pemberton; Richard Rowan-Robinson; Vidya Samadi; Will Shepherd; Kerry Sims; Simon Spooner; James Tlhomole; Chak-Hau Michael, Tso; Tim Williams; Xilin Xia

Methods

The preparation of this perspective combined 4 phases: (1) literature assessment conducted by EB and LH to define four main decision and knowledge needs in the field of water quality regulation, and the potential contributions of AI (Figure 1, main manuscript); (2) in-person, one-day workshop with invited experts; (3) preparation of a first draft of the manuscript (EB) followed by detailed review by participants to the workshop; (4) independent review by two invited AI in water experts (MG and VS) who did not participate in the workshop.

The one day workshop was held in London on April 2, 2025. The lead author (EB) identified the experts through extensive literature search and additional suggestions from LH, EP and MF. A total of 30 experts took part in the workshop, with some belonging to the same organization. Table S1 summarizes organizations represented in the workshop and the stakeholder group to which they belong. Ahead of the workshop, participants were asked to self-assign to one of four small groups based on their expertise. Groups were identified based on one of the four core decision and knowledge needs identified in Figure 1 (main manuscript).

Table S1. Organisations and stakeholder group that took part in the policy workshop, and number of participants per organisation.

Organisation	Stakeholder group	Number of participants
AtkinsRealis	Engineering consultant	1
Cambridge University	Academia	7
Centre for Ecology and Hydrology	Applied research	2
Drinking Water Inspectorate	Regulator	3
Environment Agency	Regulator	10
SciensCapital	Investor	1
Siemens	Engineering consultant	1
The Rivers Trust	Civil Society organization	1
University of Birmingham	Academia	1
University of Exeter	Academia	1
University of Oxford	Academia	1
University of Sheffield	Academia	1

The workshop combined one full-group discussion and small-group discussions. The full-group discussion was moderated by the lead author (EB) and informed by two presentations from LH and MF. For small-group discussions, moderators were appointed by each group. Note-takers (CGB, JT, SG, MGM) were previously assigned to each of the four groups and received a standard note taking template prepared by EB.

To support the small-group deliberations, EB developed a simple questionnaire to guide group discussions: (1) What are knowledge gaps and what is their policy relevance?; (2) What are AI-based research approaches or projects that might help address the identified gaps?; (3) What is the maturity of AI approaches in addressing these knowledge gaps and challenges; (4) What are key recommendations and priorities moving forward? This structure was also adopted by note-takers to report main findings from deliberations in each of the four groups.

The notes from the full-group and small-group discussions were analyzed by EB through qualitative content analysis. EB drafted the manuscript, which was reviewed iteratively by all co-authors. MG and VS reviewed the final manuscript.
